# Perceptions of Death and Religiosity Among Older Adults During the COVID-19 Pandemic in Brazil: A Qualitative Study

**DOI:** 10.7759/cureus.101305

**Published:** 2026-01-11

**Authors:** Débora de Freitas Britto Rêgo

**Affiliations:** 1 Public Health, Universidade Católica de Brasília, Brasília, BRA; 2 Geriatrics, Universidade Católica de Brasília, Brasília, BRA

**Keywords:** covid-19 pandemic, fear of death, older adults, qualitative research, religiosity

## Abstract

Introduction: The COVID-19 pandemic intensified reflections on death and fear of death, particularly among older adults, who were disproportionately affected by severe outcomes and social restrictions. Religiosity and spirituality are recognized as relevant resources for coping with existential distress; however, qualitative evidence examining these dimensions in the Brazilian context remains limited.

Objective: This study aimed to explore the subjective meanings and lived experiences of older adults regarding death, fear of death, and religiosity during the COVID-19 pandemic, comparing individuals who were infected and not infected with SARS-CoV-2.

Methods: This qualitative, descriptive, and exploratory study was conducted with 171 older adults (≥60 years) residing in Brazil. Data were collected through semi-structured interviews and analyzed using IRAMUTEQ software (Interface de R pour les Analyses Multidimensionnelles de Textes et de Questionnaires; Pierre Ratinaud, Laboratoire d’Études et de Recherches Appliquées en Sciences Sociales, Toulouse, France), reaching saturation while ensuring the mathematical stability of the lexical clusters. Analytical procedures included descending hierarchical classification (DHC), word cloud generation, and similarity analysis to identify stable multidimensional patterns in participants’ narratives.

Results: The DHC identified six stable lexical classes. Narratives highlighted the prominence of death-related themes, frequently associated with fear of suffering, family relationships, faith, and hope. Religiosity emerged as a central pillar for interpretive processes and emotional regulation. Participants who had experienced SARS-CoV-2 infection described death as a more concrete and imminent possibility, whereas non-infected participants more often emphasized anticipatory fear, vulnerability, and concern for the impact of their death on family members.

Conclusion: The findings suggest that older adults’ perceptions of death were shaped by an interrelation of emotional and spiritual factors. Religiosity functioned as a vital coping mechanism, providing a framework for acceptance and resilience. These results underscore the importance of integrating spiritual and existential dimensions into geriatric care and mental health support, especially during public health crises.

## Introduction

The COVID-19 pandemic profoundly transformed social life and intensified collective encounters with death and uncertainty. Beyond its biological impact, the pandemic produced extensive social and psychological consequences, disproportionately affecting older adults due to higher mortality rates, increased vulnerability, and prolonged periods of social isolation [1].

Early public health measures, including household isolation and social distancing, significantly altered daily routines and interpersonal relationships, contributing to emotional distress among older individuals [2]. For many older adults, the pandemic context was marked by fear of illness, fear of dying alone, and concern about losing loved ones, intensifying subjective perceptions of vulnerability and mortality [3].

Empirical evidence indicates that loneliness increased substantially among older populations during the pandemic and was strongly associated with negative mental health outcomes, including anxiety, depressive symptoms, and psychological distress [4]. Studies conducted in different sociocultural contexts highlight that social isolation exacerbated feelings of abandonment, emotional insecurity, and existential unease among older individuals [5].

COVID-19-related anxiety has been consistently associated with fear of death and psychological suffering [6,7]. Theoretical perspectives suggest that heightened mortality salience during pandemics amplifies death-related concerns, particularly in contexts marked by uncertainty, perceived lack of control, and social disconnection [7]. Intolerance of uncertainty has been identified as a relevant factor that may intensify the relationship between pandemic-related anxiety and fear of death [6].

Although social distancing measures were essential to control viral transmission, they reduced access to emotional support and disrupted coping strategies traditionally used by older adults [2,8]. In addition to emotional effects, the pandemic negatively impacted perception, memory, balance, and overall quality of life among older individuals, further contributing to psychological vulnerability [9].

At the same time, studies have shown that meaning-discovery processes, benefit-finding, and spiritual or religious beliefs may function as important resources for coping with fear, grief, and awareness of mortality during health crises [7,10]. However, qualitative evidence exploring how older adults subjectively interpret death, fear of death, and religiosity in the context of the COVID-19 pandemic, particularly in Brazil, remains limited.

Therefore, this study aimed to explore the subjective meanings and lived experiences of older adults, infected and not infected with SARS-CoV-2, regarding death, fear of death, and religiosity during the COVID-19 pandemic.

The preliminary findings of this research were presented as a poster at the II Congress of Scientific Initiation of the Catholic University of Brasília in 2023.

## Materials and methods

Study design and ethical considerations

This was a qualitative, descriptive, and exploratory study conducted in Brazil. The research aimed to explore perceptions of death, fear of death, and the role of religion among older adults during the COVID-19 pandemic. The study was approved by the Research Ethics Committee of the Universidade Católica de Brasília (CAAE No. 51397821.0.0000.0029; approval No. 4.983.852). All participants provided written informed consent prior to their inclusion, ensuring confidentiality and the voluntary nature of participation.

Study population and recruitment

The study population consisted of Brazilian older adults (≥ 60 years) residing in the Federal District, Brazil. Participants represented different religious affiliations and included individuals who had been infected and not infected with SARS-CoV-2. Recruitment was conducted using multiple strategies to ensure a diversified approach, including senior community centers in Brasília, outpatient medical clinics at the Catholic University of Brasília, and various religious institutions, supplemented by a snowball sampling strategy where initial participants referred others from their social and religious networks. Inclusion criteria required participants to be 60 years or older. Exclusion criteria included the presence of mild cognitive impairment, dementia, or any cognitive condition that could compromise comprehension of the interview questions; a medical diagnosis of psychiatric disorders; and individuals who experienced significant emotional distress when discussing the study topics.

Sample size and data saturation

The final sample size consisted of 171 participants. As the interviews progressed, the researcher observed that data saturation was achieved, characterized by the recurrence of themes and minimal emergence of new lexical patterns. However, the sample was expanded to n=171 to ensure the mathematical stability of the descending hierarchical classification (DHC) clusters. This decision was strategically made to provide a robust and high-volume textual corpus, enhancing the statistical power and reliability of the multidimensional lexical analyses performed by the IRAMUTEQ software (Interface de R pour les Analyses Multidimensionnelles de Textes et de Questionnaires; Pierre Ratinaud, Laboratoire d’Études et de Recherches Appliquées en Sciences Sociales, Toulouse, France), which benefits from larger datasets to identify significant discursive patterns.

Data collection procedures

Data collection occurred in two stages between May and August 2022, a period following the peak waves of the COVID-19 pandemic in Brazil and after the widespread vaccine rollout. This timing allowed the study to be conducted safely, enabling a hybrid approach of remote interviews via digital platforms - WhatsApp (Meta Platforms, Menlo Park, CA, USA), Google Meet (Google LLC, Mountain View, CA, USA), Zoom (Zoom Video Communications, San Jose, CA, USA), and Skype (Microsoft Corporation, Redmond, WA, USA) - and in-person interviews for those who requested face-to-face meetings or lacked digital access. Conducting the interviews during this post-peak phase enabled participants to reflect on their experiences after having lived through the most critical periods of the crisis. By this time, many had already processed emotional impacts related to loss and social isolation, allowing for more consolidated narratives regarding death and religiosity. In the first stage, participants were informed about the study objectives, and written informed consent was obtained. In the second stage, participants completed a sociodemographic and health questionnaire, followed by a semi-structured interview lasting approximately 60 minutes. All data were securely stored using an online platform (Google Forms; Google LLC), ensuring confidentiality, anonymity, and data integrity throughout the process.

Data analysis

Quantitative data were analyzed using descriptive statistics, including mean, standard deviation (±), and absolute/relative frequencies (n, %). Qualitative data analysis was performed exclusively using IRAMUTEQ, a software that supports lexical and statistical analysis of textual data. The analysis followed a systematic technical workflow starting with corpus preparation, where verbatim transcriptions were organized and standardized for software compatibility. Subsequently, a DHC was performed to identify word frequencies, lexical classes, and patterns of association within the narratives. The software facilitated the identification of central terms and their relationships, highlighting dominant discursive patterns and recurrent semantic fields related to perceptions of death, fear of death, and religiosity. IRAMUTEQ was selected due to its suitability for descriptive and exploratory research, allowing for a transparent and reproducible analytical process. To ensure interpretative validity, verbatim excerpts from the participants were selected to illustrate and support the identified lexical classes.

Terminological definition

In this study, the term “religiosity” was adopted to describe the participants' experiences. While literature often distinguishes religiosity (organized practices) from spirituality (individual search for meaning), the participants predominantly expressed their views through structured religious frameworks, such as God, faith, and prayer. As they did not explicitly differentiate these constructs in their narratives, "religiosity" was used throughout the manuscript to maintain fidelity to the participants' self-reported experiences.

## Results

A total of 171 older adults participated in the study. The sociodemographic profile, religious affiliation, and health status of the sample are summarized in Table 1. The majority of participants were female (74.3%), aged between 60 and 69 years (62%), and possessed a high level of education, with 66.1% holding a bachelor's or post-graduate degree. All participants (100%) reported a belief in God or a higher power, with Catholics (54.4%) and Evangelicals (35.1%) being the most prevalent religious affiliations. Regarding COVID-19 history, 55.6% of the participants reported having been infected with SARS-CoV-2 prior to the interview.

**Table 1:** Sociodemographic characteristics, religious affiliation, and SARS-CoV-2 infection status of the study participants (n=171). This table presents the baseline characteristics of the 171 older adults who participated in the study. All participants were residents of the Federal District, Brazil. Education levels and religious affiliations were self-reported. The SARS-CoV-2 infection status refers to whether the participant had been diagnosed with COVID-19 at any point prior to the interview (conducted between May and August 2022). n: absolute frequency; %: relative frequency

Variable	Category	Frequency (n)	Percentage (%)
Gender	Female	127	74.3
	Male	44	25.7
Age Range	60-64 years	67	39.2
	65-69 years	39	22.8
	70-74 years	28	16.4
	75-79 years	17	9.9
	80-84 years	15	8.8
	85 years or older	5	2.9
Education Level	Primary Education	14	8.2
	Secondary Education	44	25.7
	Higher Education (Bachelor's)	62	36.3
	Post-graduate Degree	51	29.8
Religious Affiliation	Catholic	93	54.4
	Evangelical	60	35.1
	Spiritist	11	6.4
	Others/Not informed	7	4.1
Belief in God/Higher Power	Yes	171	100
	No	0	0
SARS-CoV-2 Infection	Yes (Infected)	95	55.6
	No (Not infected)	76	44.4

The qualitative corpus derived from these participants was processed using multidimensional lexical analysis techniques, including DHC, word cloud generation, and similarity analysis. These complementary procedures allowed for a comprehensive understanding of the narratives regarding death, fear of death, religiosity, and the aging experience during the COVID-19 pandemic.

Word cloud analysis

**Figure 1:**
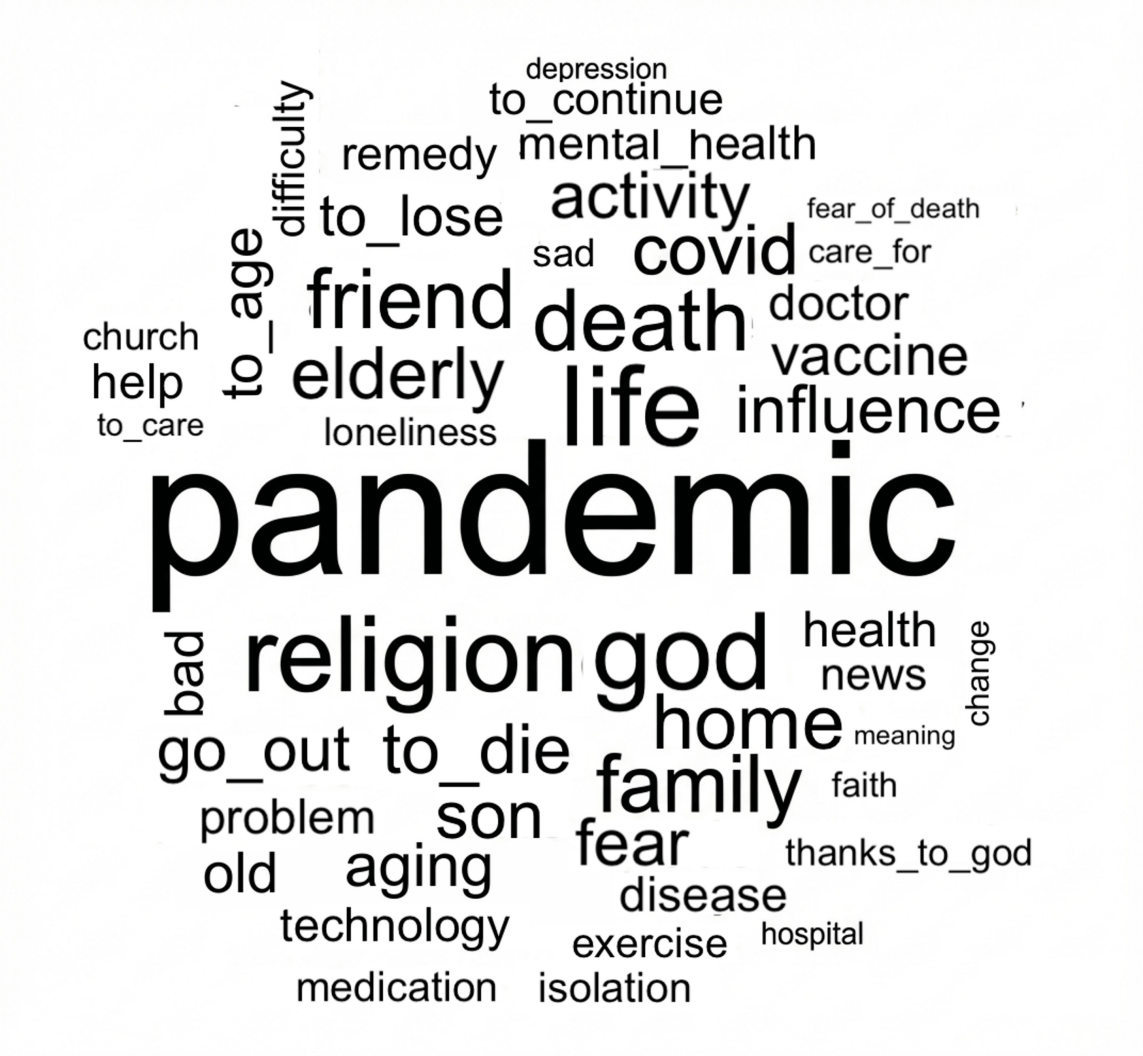
Word cloud illustrating the most frequently evoked terms by older adults during the interviews.

The word cloud analysis highlighted the most frequently evoked terms in participants’ narratives, reflecting the central discursive categories addressed during the interviews (Figure 1). The most prominent words included “pandemic” (ƒ = 1,778), “life” (ƒ = 994), “religion” (ƒ = 923), “God” (ƒ = 784), “death” (ƒ = 710), “COVID” (ƒ = 511), “vaccine” (ƒ = 469), “fear” (ƒ = 460), “aging” (ƒ = 436), and “family” (ƒ = 553).

The predominance of terms related to the pandemic context (“pandemic,” “COVID,” “vaccine”) indicates the strong influence of the health crisis on participants’ daily lives and emotional experiences. Words associated with existential dimensions (“life,” “death,” “fear”) reveal frequent reflections on mortality and fear of death, which were intensified by the widespread exposure to illness and death during the pandemic.

Religious terms such as “religion” and “God” appeared prominently, suggesting that spirituality played a central role in how older adults interpreted and coped with fear, uncertainty, and the perception of death. Additionally, the frequent mention of “family” reflects the importance of social bonds and emotional support during periods of social distancing and isolation.

Overall, the word cloud illustrates the interconnection between pandemic-related stressors, existential concerns, and coping strategies grounded in religiosity and family relationships.

Similarity analysis

**Figure 2:**
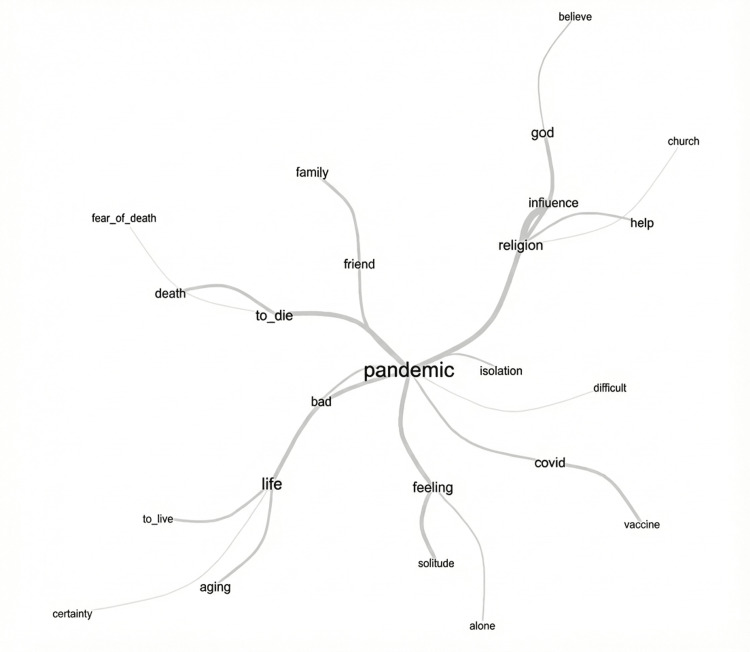
Similarity analysis illustrating the structural relationships between words within the textual corpus.

The similarity analysis explored the co-occurrence and structural relationships between words within the textual corpus, revealing how key concepts were interconnected in participants’ narratives (Figure 2).

The analysis identified “pandemic” as a central node, strongly connected to terms such as “fear,” “death,” “COVID,” “disease,” and “isolation,” indicating that the pandemic served as the primary contextual framework through which participants reflected on mortality and emotional distress. The term “death” was closely associated with words such as “fear,” “suffering,” “God,” and “acceptance,” highlighting the dual interpretation of death as both a source of anxiety and a process mediated by religious beliefs.

Another prominent cluster emerged around “religion” and “God,” which were connected to terms such as “faith,” “peace,” “hope,” and “comfort.” This structure suggests that religiosity functioned as a protective and meaning-making resource, helping older adults reinterpret death and reduce fear associated with uncertainty and loss.

The term “family” formed an additional cluster linked to “care,” “support,” and “home,” reflecting the emotional significance of familial relationships during periods of social isolation. Together, these networks demonstrate that participants’ perceptions of death were not isolated constructs but embedded within broader emotional, social, and spiritual contexts shaped by the pandemic.

Descending hierarchical classification (DHC)

**Figure 3:**
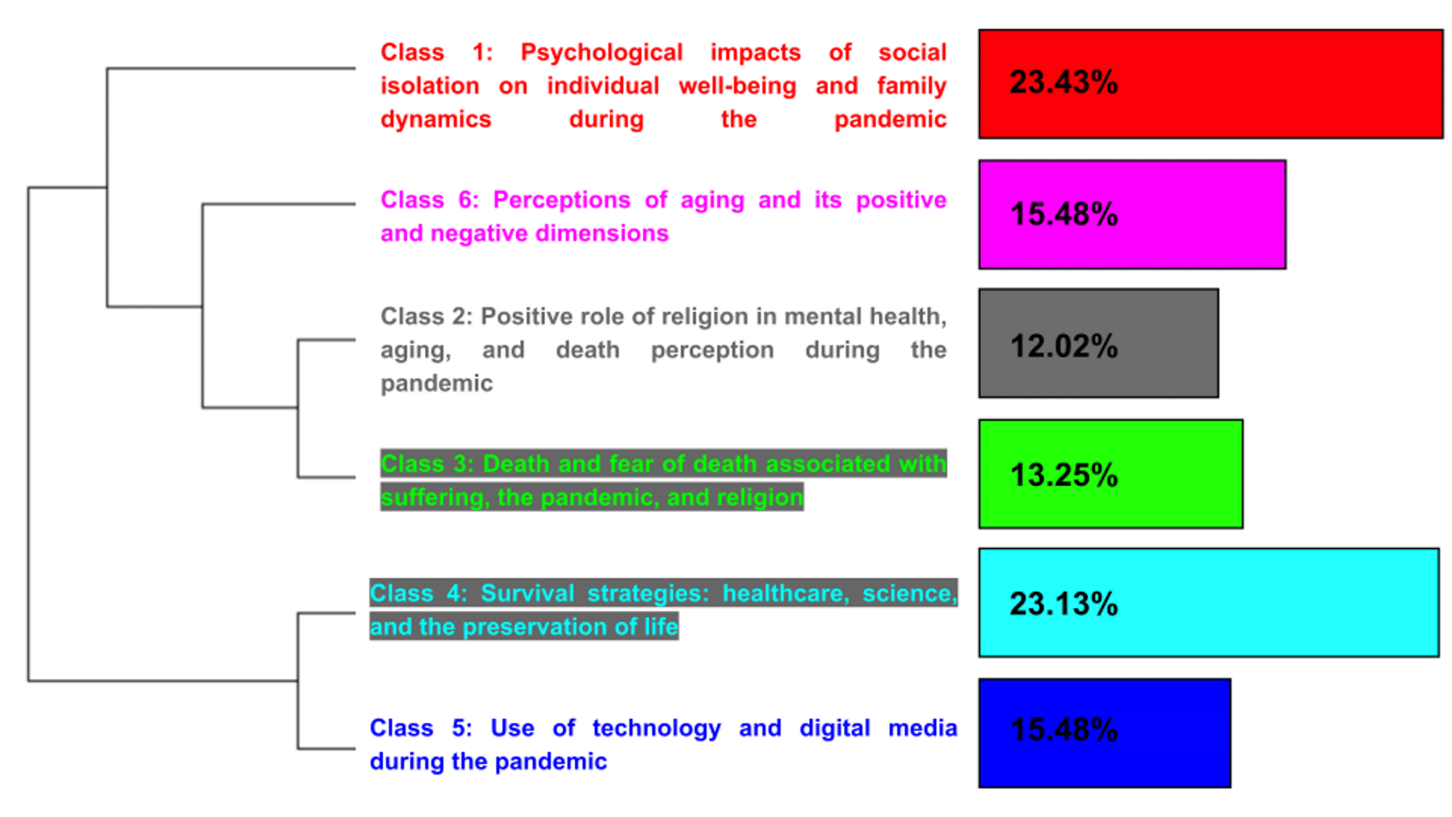
Descending hierarchical classification dendrogram of the interview corpus.

The DHC divided the corpus into six stable lexical classes (Figure 3), reflecting distinct but interconnected dimensions of participants’ experiences.

Class 1: Psychological Impacts of Social Isolation on Individual Well-Being and Family Dynamics

This class represented 23.43% (f = 1,547 STs) of the corpus. Participants described emotional suffering associated with prolonged social distancing, including feelings of loneliness, anxiety, and sadness due to reduced social contact. Many narratives emphasized the disruption of daily routines and the loss of face-to-face interactions. Conversely, some participants reported positive experiences, noting that staying at home strengthened family bonds, increased feelings of protection, and provided emotional security. Representative excerpts from participants’ narratives include:

“We had to isolate and distance ourselves from family members, people could not go out much, and that made me feel depressed. I felt lonely during the pandemic.” (Participant 67, Female, 60-65 years)

“There was a period at the beginning of the lockdown when almost everyone stayed at home, and I actually liked it because I spent more time with my wife and my children.” (Participant 106, Male, 60-65 years)

Class 2: Positive Role of Religion in Mental Health, Aging, and Death Perception

Accounting for 12.02% (f = 794 STs), this class highlighted religion as a key resource for emotional regulation and existential meaning. Participants described faith as a source of comfort, inner peace, and resilience in the face of uncertainty, illness, and fear of death. Religious beliefs facilitated acceptance of aging and death, with several participants expressing trust in a divine plan and belief in continuity beyond physical life. This class underscores the protective role of spirituality in mental health during the pandemic. Representative excerpts illustrate the perceived protective role of religion:

“The role of religion in my mental health is the faith we have in God, which makes us believe that we can stay calm and that everything will work out.” (Participant 67, Female, 60-65 years)

“Religion does influence my perception of aging; I need to believe in God in order to see meaning in life.” (Participant 147, Female, 60-65 years)

Class 3: Death and Fear of Death Associated With Suffering, the Pandemic, and Religion

This class comprised 13.25% (f = 875 STs) of the corpus and focused explicitly on death-related perceptions. Participants discussed death as a natural and inevitable event, often contextualized within religious frameworks that reduced fear and promoted acceptance. However, fear of death persisted, particularly fear of suffering, dying alone, and leaving family members behind. Media exposure and frequent reports of COVID-19-related deaths intensified awareness of mortality and contributed to emotional distress. Representative excerpts highlight different ways participants perceived death and fear of death:

“Death itself is just a passage; it is through religion that I see it this way. That is why I am not afraid of death-I am afraid of suffering.” (Participant 27, Female, 66-70 years)

“My perception of death changed during the pandemic because I became worried about getting sick, being confined to a bed to die, and suffering at the moment of death.” (Participant 67, Female, 60-65 years)

Class 4: Survival Strategies: Healthcare, Science, and the Preservation of Life

This class (23.13%, f = 1,527 STs) articulates how participants sought to preserve life through science. The decision to vaccinate was seen as a rational act of self-preservation, often independent of religious dogma, reflecting a "science-faith" coexistence. Participants in this class described personal or vicarious experiences with COVID-19, emphasizing fear of infection, uncertainty regarding disease progression, and concerns about health vulnerability due to aging. Representative excerpts illustrate participants’ decisions regarding vaccination:

"My spirituality did not influence my decision to get vaccinated; vaccination is science and science is what saves us from death." (Participant 147, Female, 60-65 years)

"I took the vaccine as soon as possible because I wanted to live longer; I followed all health protocols to avoid the virus." (Participant 15, Female, 66-70 years)

Class 5: Use of Technology and Digital Media During the Pandemic

Representing 12.69% (f = 838 STs), this class addressed the use of digital tools for communication, religious activities, and social interaction. Participants reported that technology enabled continued contact with family, friends, and religious communities. Despite these benefits, difficulties such as limited digital literacy, lack of access to devices, and discomfort with technology were also reported, highlighting digital exclusion as a relevant challenge among older adults. Representative excerpts illustrate patterns of technology use:

"I used the phone to watch the masses online; it was the only way to keep my faith active while the churches were closed." (Participant 149, Female, 76-80 years)

"WhatsApp was my salvation to see my grandchildren's faces; without this technology, the loneliness would have been unbearable." (Participant 56, Male, 60-65 years)

Class 6: Perceptions of Aging and Its Positive and Negative Dimensions

This class accounted for 15.48% (f = 1,022 STs) of the corpus and explored how participants perceived the aging process. Aging was associated with physical limitations, health concerns, and fear of dependency. At the same time, participants emphasized positive aspects such as accumulated life experience, emotional maturity, wisdom, and increased spirituality. This class demonstrates that aging was perceived as a multifaceted process, shaped by both vulnerability and resilience, especially within the context of the pandemic. Representative excerpts illustrate participants’ ambivalent perceptions of aging:

“I see aging as something normal-it is a natural process of life: being born, growing up, aging, and dying. There are positive and negative aspects.” (Participant 90, Female, 60-65 years)

“For me, aging has both a positive and a negative side. Younger people often do not see us as capable, and what we say is not valued.” (Participant 87, Female, 60-65 years)

## Discussion

The present qualitative study investigated perceptions of death, fear of death, aging, and religiosity among older adults during the COVID-19 pandemic. By integrating lexical analyses, including word cloud visualization, similarity analysis, and DHC, the study provides an exploratory overview of how older adults articulated existential concerns, emotional experiences, and coping-related narratives within a context of prolonged uncertainty and social disruption.

The prominence of pandemic-related terms in the word cloud analysis underscores COVID-19 as a central organizing element in participants’ narratives. This pattern is consistent with evidence indicating that the pandemic intensified collective awareness of mortality, particularly among older adults, who were disproportionately affected by severe outcomes and mortality rates [1]. The frequent co-occurrence of death-related terms with emotional expressions suggests a substantial psychological burden associated with continuous exposure to illness, loss, and uncertainty during the pandemic [6,7].

The similarity analysis revealed strong lexical connections between concepts such as death, fear, loneliness, and religiosity, indicating that references to mortality were embedded within broader psychosocial and spiritual contexts. Heightened fear of death during the pandemic has been widely documented and is often associated with uncertainty, loss of control, and social isolation [6,7]. In this study, fear-related narratives extended beyond death itself to include concerns about suffering, dying alone, and the well-being of loved ones, which aligns with previous findings that pandemic-related fear encompassed both personal and relational dimensions [3].

Social isolation emerged as a salient dimension across the lexical analyses and was particularly evident in one of the main classes identified through DHC. Participants frequently referenced loneliness, sadness, and emotional distress related to reduced social contact, corroborating studies demonstrating that distancing measures significantly affected older adults’ mental health during the pandemic [2,4]. These findings are consistent with evidence that loneliness among older populations was associated with anxiety, psychological distress, and reduced well-being [4].

At the same time, participants’ narratives reflected an ambivalent experience of isolation. While physical distancing was associated with emotional suffering, some participants described isolation as a protective strategy and reported strengthened family relationships. This duality is consistent with prior research showing that although isolation disrupted daily routines and social support, family bonds sometimes functioned as protective factors, mitigating psychological distress among older adults [2,5].

Religiosity and spirituality emerged prominently in narratives related to coping with death and uncertainty, particularly when death was framed as a natural life transition rather than solely as a source of fear. These findings are consistent with empirical and theoretical literature suggesting that spiritual beliefs may help individuals manage death-related anxiety by supporting meaning attribution, emotional regulation, and existential coherence [7,10]. Within this exploratory context, religiosity appeared to operate as a psychological and emotional resource rather than as a determinant of specific outcomes.

Another lexical class highlighted concerns related to health vulnerability and fear of COVID-19 infection. Participants frequently expressed anxiety regarding disease severity and outcomes, particularly in light of age-related health risks. This pattern aligns with evidence indicating that COVID-19-related anxiety is associated with fear of death and psychological distress, especially when combined with intolerance of uncertainty [6,9]. Similar patterns have been observed in other populations exposed to sustained stress during the pandemic, suggesting that heightened mortality awareness was not exclusive to older adults [11]. These perceptions of vulnerability contributed to persistent emotional distress and reinforced reflections on fear of death.

Participants’ narratives also reflected the broader impact of social distancing on emotional and functional well-being. Previous studies have reported that pandemic-related restrictions negatively affected cognition, balance, emotional health, and overall quality of life among older adults [8]. References to emotional fatigue, reduced activity, and functional limitations in the present study are consistent with these findings, although causal inferences cannot be drawn.

Grief and anticipatory loss were also evident in participants’ accounts, particularly in references to the deaths of acquaintances and concerns about losing family members. Disruptions in mourning rituals and reduced opportunities for collective grieving have been shown to intensify emotional distress among older adults during the pandemic [12]. Such experiences may have contributed to heightened awareness of mortality and emotional vulnerability during this period.

Overall, the findings suggest that older adults’ perceptions of death during the COVID-19 pandemic were shaped by a complex interaction of fear, social isolation, perceived vulnerability, and processes of meaning attribution. While uncertainty and isolation emerged as significant stressors, references to religiosity, family relationships, and adaptive interpretations of aging and death appeared to function as important supportive elements within participants’ narratives. These results reinforce the relevance of holistic approaches to older adult care during public health crises, including attention to psychological, social, and spiritual dimensions.

This study has limitations that should be acknowledged. As a qualitative investigation with a geographically restricted sample, the findings are not intended to be generalized to all older adult populations. Data were derived from self-reported narratives and may be influenced by recall bias, emotional state at the time of the interview, and social desirability. Additionally, although remote interviews enabled safe data collection during the pandemic, this format may have limited participation among individuals with restricted access to digital technologies or lower digital literacy. Despite these limitations, the exploratory qualitative approach allowed for a detailed examination of subjective meanings and lived experiences related to death during the COVID-19 pandemic.

Theoretical framework and interpretive anchoring

The findings of this study can be theoretically anchored in established psychological and gerontological models that address mortality awareness, the construction of meaning, and religious coping.

First, Terror Management Theory (TMT) provides a useful framework for understanding the heightened salience of death observed during the COVID-19 pandemic. According to TMT, awareness of mortality generates existential anxiety, which individuals manage through cultural worldviews, social connections, and belief systems that provide meaning and symbolic immortality [13,14]. In the present study, frequent references to fear of death, suffering, vulnerability, and dying alone reflect classic mortality salience processes intensified by prolonged exposure to pandemic-related uncertainty. Religiosity and close family relationships appeared to function as protective buffers against death-related anxiety, consistent with TMT propositions [13].

Second, the findings are consistent with the Meaning-Making Model, which posits that individuals confronted with stressful or life-threatening events actively seek to restore coherence between global beliefs and situational experiences [15]. Participants’ narratives revealed efforts to reinterpret death, aging, and pandemic-related losses in ways that preserved meaning, emotional balance, and continuity of self. Acceptance of death as a natural life stage, reflections on aging as a process of learning and maturation, and reinterpretation of suffering within a broader existential framework illustrate adaptive existential re-evaluation processes among older adults [15,16].

Finally, the results strongly resonate with Pargament’s Religious Coping Framework, which conceptualizes religion as a multidimensional coping resource used to manage stress, uncertainty, and existential threats [17]. Positive religious coping strategies, such as trust in God, belief in life after death, and engagement in spiritual practices, were prominent in participants’ accounts and were associated with emotional comfort, resilience, and reduced fear of death. These findings suggest that religiosity functioned not only as a belief system but also as an active coping mechanism during the pandemic, supporting psychological adjustment amid social isolation, loss, and health-related fears [17,18].

Anchoring the results within these theoretical models enhances the interpretive depth of the study by demonstrating that older adults’ perceptions of death and aging during the COVID-19 pandemic extend beyond descriptive experiences and reflect broader psychological mechanisms related to mortality awareness, meaning construction, and religious coping.

## Conclusions

The findings of this qualitative study, derived from a robust textual corpus (n=171), suggest that older adults’ perceptions of death during the COVID-19 pandemic were shaped by a complex interrelation of fear, social isolation, and reflections on mortality. The lexical analyses performed by IRAMUTEQ identified stable discursive patterns where death-related themes were deeply embedded in participants’ emotional narratives, reflecting the psychological impact of prolonged uncertainty and exposure to loss. Religiosity emerged as a central pillar of sense-making, functioning as a source of emotional comfort and a symbolic buffer against death-related anxiety. In line with established psychological frameworks, religious beliefs did not necessarily eliminate the fear of suffering or loneliness, but rather coexisted with these concerns, offering a structured worldview through which participants could articulate acceptance and hope. This study confirms that for this population, religiosity is not merely a set of rituals but a vital coping mechanism for managing existential distress during public health crises.

Taken together, these results underscore the importance of integrating psychological, social, and spiritual dimensions into geriatric care. The findings highlight the need for supportive interventions that include mental health care, opportunities for social connection, and sensitivity to the existential concerns of older adults. Future research should continue to explore these dimensions to further clarify the long-term impacts of the pandemic on aging, vulnerability, and the construction of meaning in the face of mortality.
